# Supplementary material for: Regulation of Cortico-Thalamic JNK1/2 and ERK1/2 MAPKs and Apoptosis-Related Signaling Pathways in PDYN Gene-Deficient Mice Following Acute and Chronic Mild Stress
Source: Int J Mol Sci. 2023 Jan 24;24(3):2303. doi: 10.3390/ijms24032303 (PMC9916432; doi:10.3390/ijms24032303)
Supplement: Supplementary file 1 [file ijms-24-02303-s001.zip › ijms-2109079-SI.pdf]

Supplementary Table S1. Contingency table summarizing the distribution of mice across the experimental groups of the study

Stress procedure	Genotype		Total
	WT	PDYN-KO	
Basal stress	9	9	**18**
Acute restrain stress (ARS)	8	7	**15**
Chronic mild stress (CMS)	8	7	**15**
**Total**	**25**	**23**	**48**
